# Cell-bound IgE and plasma IgE as a combined clinical diagnostic indicator for allergic patients

**DOI:** 10.1038/s41598-020-61455-8

**Published:** 2020-03-13

**Authors:** Chuanghua Qiu, Lihong Zhong, Chunxiu Huang, Jia Long, Xuejun Ye, Jingbo Wu, Wenjie Dai, Wei Lv, Chongwei Xie, Junfang Zhang

**Affiliations:** 1 Shenzhen Second People’s Hospital, First Affiliated Hospital of Shenzhen University, College of Life Sciences and Oceanography Shenzhen University, Shenzhen, Guangdong 518035 China; 2 Shenzhen Immunotherapy Biotechnology Co., Ltd, Shenzhen, Guangdong 518109 China; 3 Medical Research Center, Yuebei People’s Hospital, Shaoguan, Guangdong 512026 China; 4 Shenzhen University General Hospital, Shenzhen, Guangdong 518035 China

**Keywords:** Medical research, Epidemiology

## Abstract

Allergic responses are mainly caused by IgE, which is often located on the cell surface. The current diagnostic method detects both allergen-specific IgE and total IgE levels, but a number of allergic patients have a normal serum IgE level, which is a poor clinical correlate for allergy. Here, we developed a simple method to detect the level of cell-bound IgE by dissociating it from blood cells with lactic acid. Dissociated cell-bound IgE and plasma IgE levels were detected using the same ELISA kit at the same time. We established two clinical cohorts: an allergic patient group and a healthy participant group. In general, cell-bound IgE correlated well with plasma IgE; however, some patients exhibited high cell-bound IgE levels but low plasma IgE levels. We recommended 350 ng/mL peripheral blood total IgE (cell-bound IgE + plasma IgE) as the cut-off value for allergy diagnosis. Using this indicator, 90.32% of our allergic patients were correctly diagnosed. The peripheral blood total IgE level is a promising clinical diagnostic indicator in allergic patients and will provide more guidance for allergy diagnosis and therapeutic evaluation.

## Introduction

Immunoglobulin E (IgE) plays a key role in the development of allergic diseases^1,2^, and it is necessary to detect the IgE level for diagnosis and treatment evaluation^3^.

The concentration of IgE in the circulation is very low (below 240 ng/ml in healthy individuals); it is the least prevalent antibody type, with a level much lower than the normal level of IgG (5–10 mg/ml)^4^. The half-life of free IgE in the blood is only 2–3 days, while IgE bound to the high-affinity receptor FcεRI on mast cells or basophils is stable for several weeks^5^. Most IgE is bound to cells through its receptors, leaving only a small proportion free in the plasma^6^. Serum IgE levels are very important for the diagnosis of allergies and generally correlate with disease severity^7^. However, the clinical detection of IgE is limited to free serum/plasma IgE, which ignores the large contribution of cell-bound IgE^8^. A number of allergic patients have normal serum IgE levels, which is why the World Allergy Association does not recommend the use of total IgE as a diagnostic guideline for allergy^9^. The level of free IgE in the blood is usually measured by ImmunoCAP^10^.

Allergen-specific IgE is the causative agent of allergic disease. Several studies have reported that specific IgE levels correlate well with the severity of allergy; however, a relatively high number of molecules must be defined and produced at a sufficient quality to cover all clinically important allergen specificities^11^. Not all allergens that are in extracts have been defined at the molecular level yet. Other allergens have been well characterized but have not been produced at the quality level required for component-resolved diagnostic tests. The skin prick test is the gold standard for diagnostic allergy tests and is used to confirm allergic sensitization to suspected allergens and provide guidance for the treatment of patients. While this test can be uncomfortable for patients, it also has an occasional risk of infection, though it is relatively safe.

There are two IgE receptors: FcεRI and FcεRII. FcεRI is a high-affinity receptor that is ubiquitous on mast cells and basophils. Other types of cells, such as dendritic cells, Langerhans cells, monocytes/macrophages, eosinophils, and platelets, have lower FcεRI expression^7^. FcεRII, also known as CD23, is a low-affinity receptor. Studies have shown that CD23 is expressed on B cells, monocytes, T cells, dendritic cells, platelets and neutrophils^8^. Eleonora Dehlink *et al*. investigated the relationships among the levels of serum IgE, cell-bound IgE, and IgE receptors in a pediatric population. The authors analyzed FcεRI, CD23 and cell-bound IgE on peripheral blood cells by flow cytometry, measured total serum IgE concentrations by ELISA and found that cell-bound IgE on FcεRI^+^ cells correlated well with serum IgE generally; however, some patients exhibited high amounts of cell-bound IgE but low total serum IgE levels^11,12^.

Soluble IgE receptors may mediate IgE-mediated immune responses and are important in the understanding of allergic responses. Soluble FcεRI (sFcεRI) is found as a soluble free IgE receptor and in complex with IgE. An association between sFcεRI and allergy severity has been reported, but some individuals with normal IgE levels have high levels of sFcεRI^13,14^. The density of CD23 molecules on B cells has been shown to correlate with total IgE levels^8^. In addition to its transmembrane forms, CD23 has been found as a soluble protein in human serum, and several reports have shown that the severity of allergy correlates directly with serum levels of soluble CD23 (sCD23)^15,16^. However, CD23 is approved as a prognostic parameter only for B-cell chronic lymphocytic leukemia^17^.

Here, we developed a new and simple method for determining cell-bound IgE levels. We collected samples from 93 allergic patients and 102 healthy people and analyzed the relationship between serum IgE levels and cell-bound IgE levels to provide more guidance for allergy diagnosis and therapeutic evaluation.

## Methods

### Study population

Two clinical cohorts were established: an allergic patient group (n = 93) and a healthy participant group (n = 102). Patient personal information, such as age, sex and clinical diagnosis, was obtained. We obtained peripheral blood samples for flow cytometry analysis and IgE level measurements by ELISA at the time of enrollment.

The inclusion criteria for the allergic patients depended on the doctors’ clinical diagnosis and confirmation of the allergy information and allergic symptoms in clinical diagnostic reports at the Shenzhen Second People’s Hospital. The patients selected as allergic individuals had a positive CAP result in a test with 21 different allergen extracts.

We obtained information on the allergic symptoms of the patients from the clinical diagnostic reports at the hospital. Personal information, such as age and sex, was collected through a questionnaire, and written informed consent was provided by the patients.

The project was approved by the ethical committee of the Shenzhen Second People’s Hospital (No. 046), which voted unanimously for its completion, and conformed to the basic principles of medical ethics. All the methods were performed in accordance with the relevant guidelines and regulations.

### Cell-bound and plasma IgE quantitative determination

Fresh whole blood from allergic patients and healthy people was collected with EDTA as the anticoagulant. One milliliter of whole blood from each sample was used after centrifugation at 500 g for 15 min (all the following centrifugations were performed at 500 g for 15 min), and the upper plasma layer was collected. Then, 1 mL of ACK lysis buffer (Thermo Fisher Scientific, USA) was added to the cell precipitate on ice for 10 min to lyse the red blood cells. After centrifugation, the cell precipitate, which contained all white blood cells (WBCs), was collected, and 0.5 mL of lysis buffer (10 mmol/L lactic acid, 130 mmol/L NaCl, 5 mmol/L KCl, pH 3.5) was added. The samples were then vortexed for 5 seconds, followed by an incubation on ice for 5 min to dissociate IgE from the cell membrane. The samples were then centrifuged, and the supernatant was removed for renaturation with 0.5 mL of 5 mM NaOH, resulting in a total volume of 1 mL. The collected plasma and cell-bound IgE were used for IgE quantitative determination using the Elecsys IgE II Immunoassay (Roche Diagnostics GmbH, Germany). The IgE concentrations in the healthy, nonatopic test subjects were greatly dependent on age, and the recommended threshold value for adults is 240 ng/mL.

### Airborne and food allergen test

The EUROLINE test kit (EUROIMMUN, USA) was used as a semiquantitative *in vitro* assay for the detection of human IgE antibodies to airborne and food allergens in the serum or plasma. The test kit contains test strips coated with parallel lines of 21 different allergen extracts. The test strips were first moistened and incubated with patient serum in the first reaction step. Positive samples showed specific IgE antibodies binding to the allergens. The readout for the EUROLINE kit is shown in Supplementary Table S1.

### Flow cytometry

The antibodies used for flow cytometry were mouse anti-human IgE (clone G7–26, BD Biosciences, USA), mouse anti-human CD23 (clone M-L233, BD Biosciences, USA), and mouse anti-human FcεRI (clone AER-37, BD Biosciences, USA). Flow cytometric analysis was performed on a FACSCalibur (BD Biosciences, San Jose, CA, USA). Forward scatter (FSC) and side scatter (SSC) signals were recorded in the linear mode, and fluorescence signals were recorded in the logarithmic mode. Data were analyzed using CellQuest software (BD Biosciences, USA).

### Statistical analysis

Data were analyzed with SPSS for Windows (version 16.0, SPSS Inc., Chicago, IL).

A paired t-test was used to determine statistical significance. A p-value less than 0.05 was considered significant.

## Results

### Serum IgE levels do not correlate well with allergy diagnosis

**Figure 1:**
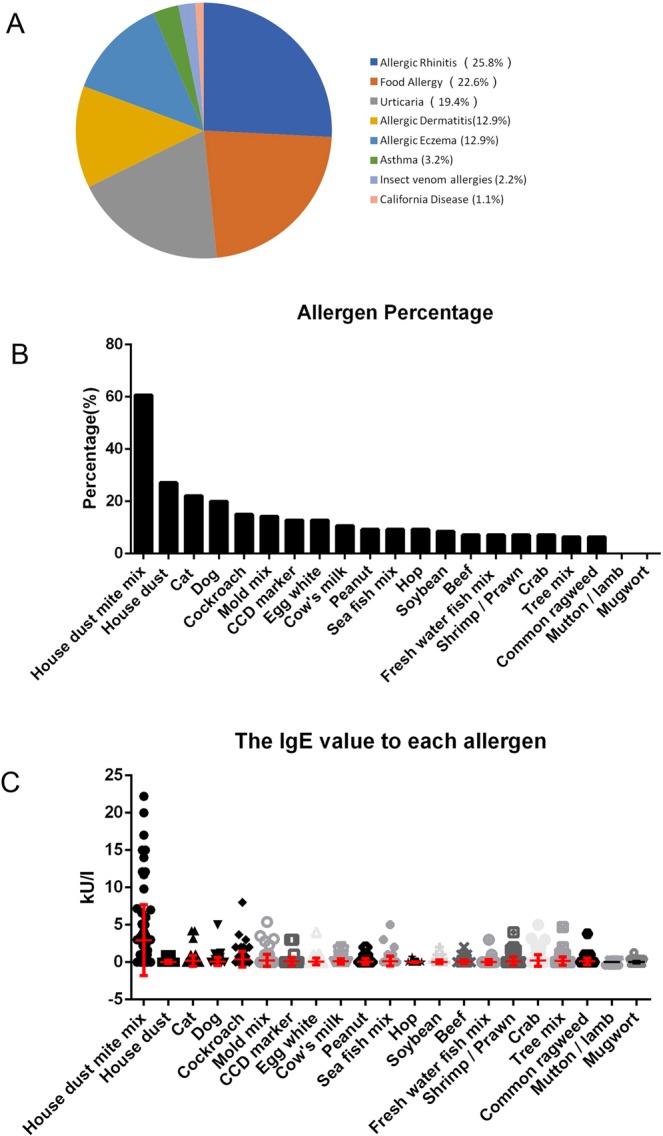
Allergic disease types and allergen identification. (**A**) The allergic disease types of all allergic patients; (**B**) the EUROLINE test kit was used to detect human IgE antibodies to 21 different airborne and food allergens in the serum of the allergic patients; and (**C**) the IgE level for each allergen was detected, and statistical analysis is shown.

Two clinical cohorts were established as shown in Table 1: the allergic patient group (n = 93; 45 males and 48 females; mean age of 29.2 ± 5.2 years) and the healthy group (n = 102; 50 males and 52 females; mean age of 27.5 ± 4.3 years). Only 34.4% of the allergic patients had elevated serum IgE levels; instead, most of the patients had normal serum IgE levels, indicating that clinical serum IgE levels are a poor clinical correlate for allergy diagnosis. We analyzed the allergic disease types of all allergic patients (Fig. 1A) and found that allergic rhinitis (25.8%), food allergy (22.6%), urticaria (19.4%), allergic dermatitis (12.9%), and allergic eczema (12.9%) were the main types of allergic diseases. A small number of people were diagnosed with asthma (3.2%), insect venom allergy (2.2%) or California disease (1.1%). The symptoms of the allergic patients are shown in Table S2.

**Table 1:** Study population.

Variable	Allergic patients			Healthy people		
	All	Normal IgE	Elevated IgE	All	Normal IgE	Elevated IgE
Subjects, n (%)	93	61 (65.6)	32 (34.4)	102	99 (97.0)	3 (3.0)
Age, mean ± SD	29.2 ± 5.2	28.3 ± 4.8	29.8 ± 6.3	27.5 ± 4.3	28.2 ± 4.5	27.2 ± 5.2
Sex						
Male, n (%)	45 (48.4)	33 (73.3)	12 (26.7)	50 (49.0)	49 (98.0)	1 (2.0)
Female, n (%)	48 (51.6)	28 (58.3)	20 (41.7)	52 (51.0)	50 (96.2)	2 (3.8)

The recommended threshold value of the IgE concentration in adults is 240 ng/mL.

To learn more about the allergic patient group, we used the EUROLINE test kit to detect human IgE antibodies specific for 21 different airborne and food allergens in allergic patient serum. The readout for the EUROLINE kit is shown in Supplementary Table S1. As shown in Fig. 1B, dust mite was the main allergen, and 60.7% of the population had IgE antibodies specific for this allergen. Other common allergens, such as house dust (27.1%), cat (22.1%), dog (20%), cockroach (15%) and mold (14.3%), were also common in this population. The IgE value for each allergen was detected, and statistical analysis of the data is shown in Fig. 1C. We also confirmed that the healthy people were not allergic to any of the allergens (data not shown).

### Allergic patients have relatively high cell-bound IgE levels

**Figure 2:**
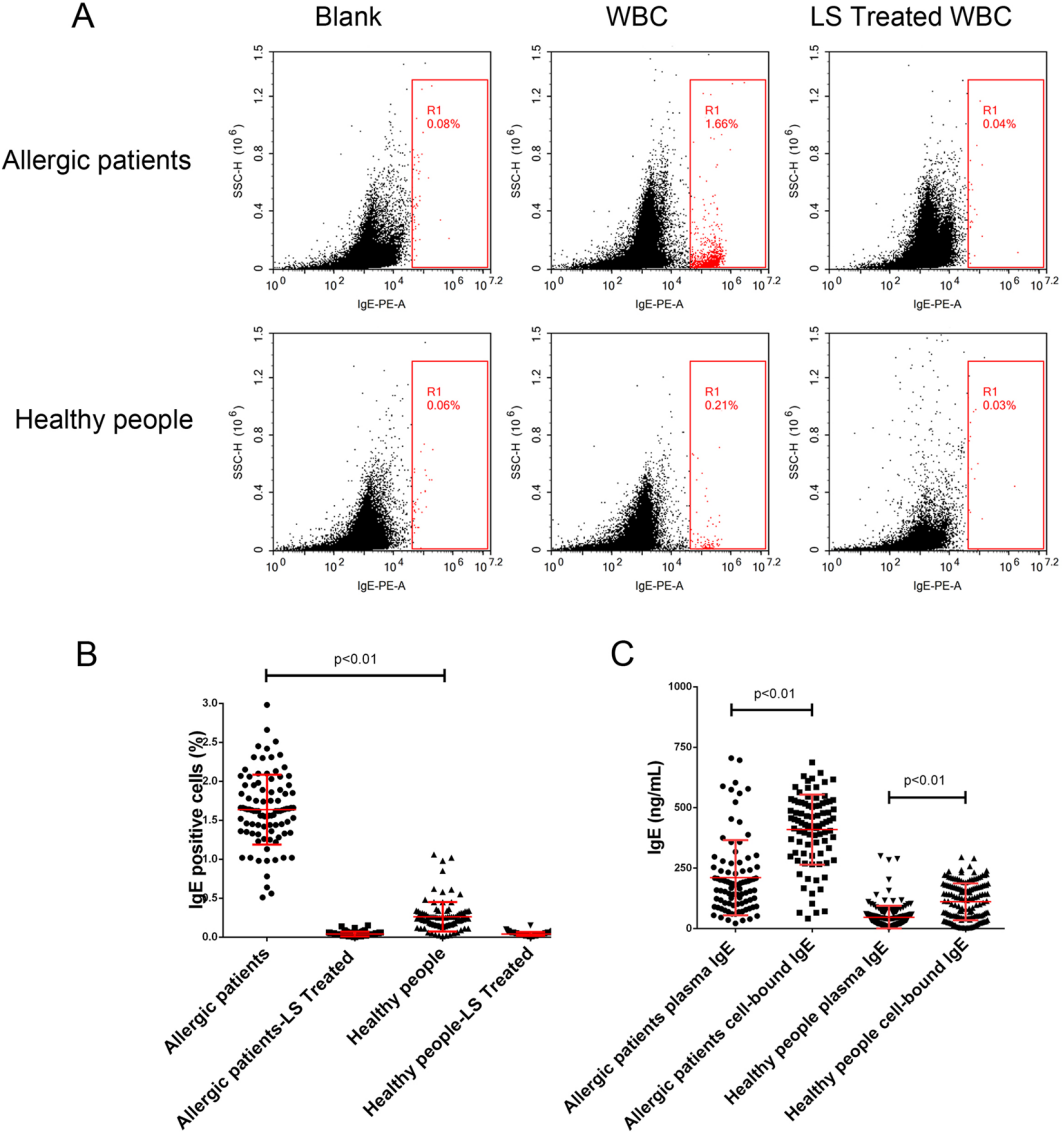
Cell-bound IgE detection. (**A**) To confirm that all cell-bound IgE was dissociated from the cell membrane, anti-IgE PE-conjugated antibody was used for staining before or after lysis buffer treatment, flow cytometry was performed to analyze the IgE+ cell percentages in WBCs; (**B**) IgE+ cells in allergic patients and healthy people were evaluated before and after lysis buffer treatment; and C, we detected the dissociated cell-bound IgE and plasma IgE levels by ELISA.

We developed a new and simple method for detecting cell-bound IgE: whole blood was first centrifuged to separate peripheral blood cells from the plasma, red blood cells were lysed with ACK buffer to obtain WBCs, and cell-bound IgE was dissociated from its membrane receptors. The collected plasma and cell-bound IgE were used for IgE quantitative determination using an ELISA kit. To confirm that all cell-bound IgE was dissociated from the cell membrane, we used anti-IgE PE-conjugated antibody staining and flow cytometry to analyze WBCs. As shown in Fig. 2A, among all the WBCs of the allergic patients, 1.66% were IgE positive, whereas only 0.04% of WBCs were IgE positive after lysis buffer treatment, which was even lower than the percentage in the blank sample (unstained). The data indicated that all cell-bound IgE was dissociated from the cell membrane after lysis buffer treatment. We quantified the percentage of WBCs with cell-bound IgE in all of the samples and found that the allergic patients had significantly higher cell-bound IgE levels than the healthy people. Lysis buffer treatment was able to dissociate cell-bound IgE from the cell membrane (Fig. 2B).

We compared the cell-bound IgE level with the plasma IgE level in each group and observed that the cell-bound IgE level was significantly higher than the plasma IgE level in the allergic patient group (p < 0.01). Consistent results were also found in the healthy participant group (Fig. 2C). The data indicated that most of the IgE was bound to the cell surface.

### Cell-bound IgE levels are a better clinical diagnostic indicator than plasma IgE levels

**Figure 3:**
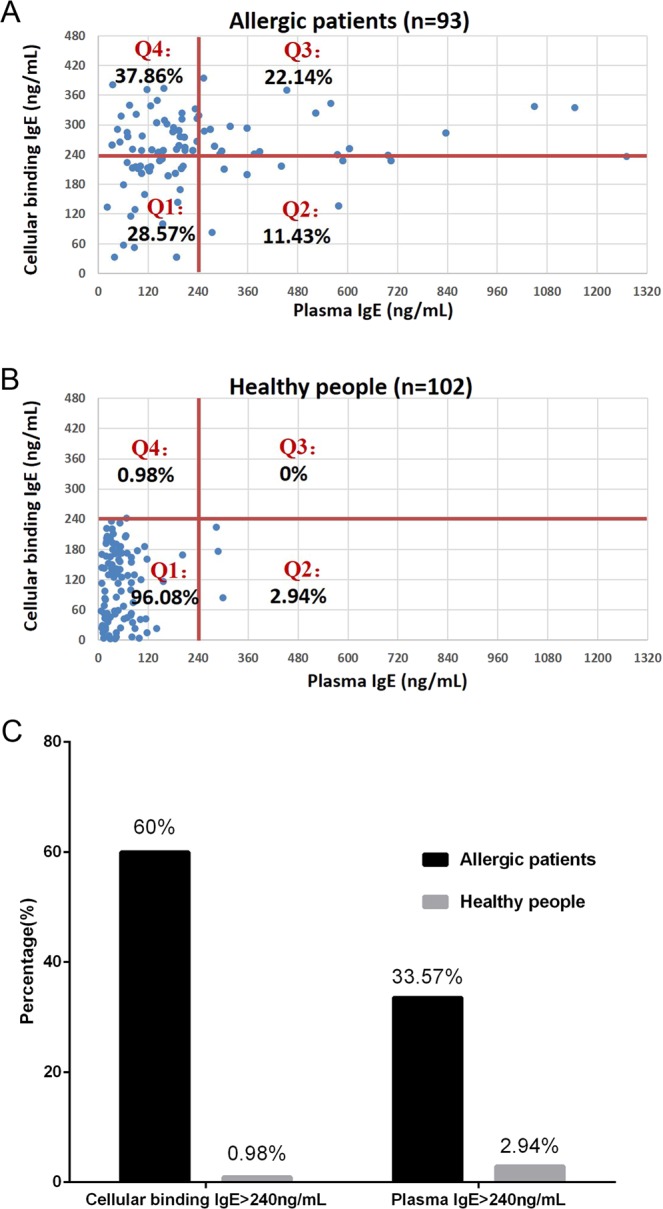
Comparison of cell-bound IgE and plasma IgE levels. The X-Y graph in which each point represents a sample, the X axis indicates the plasma IgE level, and the Y axis indicates the cell-bound IgE level. Two cut-off lines was drew at 240 ng/ml for both plasma IgE and cell-bound IgE, resulting in four quadrants (Q1–Q4). The percentage of cells in each quadrant is shown in the figure. (**A**) Plasma IgE and cell-bound IgE in the allergic patient group; (**B**) plasma IgE and cell-bound IgE in the healthy group; and (**C**) the percentages of allergic patients and healthy people diagnosed with allergy using 240 ng/ml plasma IgE or 240 ng/ml cell-bound IgE as the cut-off value.

To illustrate the relationship between the cell-bound IgE and plasma IgE levels, we created an X-Y graph in which each point represents a sample with the X axis representing the plasma IgE level and the Y axis representing the cell-bound IgE level (Fig. 3A,B). Per the kit instructions, we used a cut-off value of 240 ng/ml to draw two cut-off lines, one for plasma IgE and one for cell-bound IgE, resulting in four quadrants (Q1–Q4). As shown in Fig. 3C, in the allergic patient group, only 33.57% (Q2 + Q3) of the allergic patients had a plasma IgE level >240 ng/ml; however, 60.00% (Q3 + Q4) of these patients had a cell-bound IgE level >240 ng/ml. If the current clinical diagnostic standard (240 ng/ml plasma IgE level) was used, only 33.57% of the allergic patients met the criterion for the diagnosis of an allergy. However, if 240 ng/ml cell-bound IgE was used as the cut-off value, 60.00% of the allergic patients met the criteria for an allergy diagnosis (Fig. 3C).

Furthermore, in the population of allergic patients with a plasma IgE level >240 ng/ml (Q2 + Q3), 65.95% (Q3/(Q2 + Q3)) had a cell-bound IgE level >240 ng/ml. Among the healthy participants, 2.94% (Q2 + Q3) had a plasma IgE level >240 ng/ml, and only 0.98% (Q3 + Q4) had a cell-bound IgE level >240 ng/ml. Therefore, the cell-bound IgE level is a better clinical diagnostic indicator of allergy than the plasma IgE level.

### Peripheral blood total IgE is recommended for allergy diagnosis

**Figure 4:**
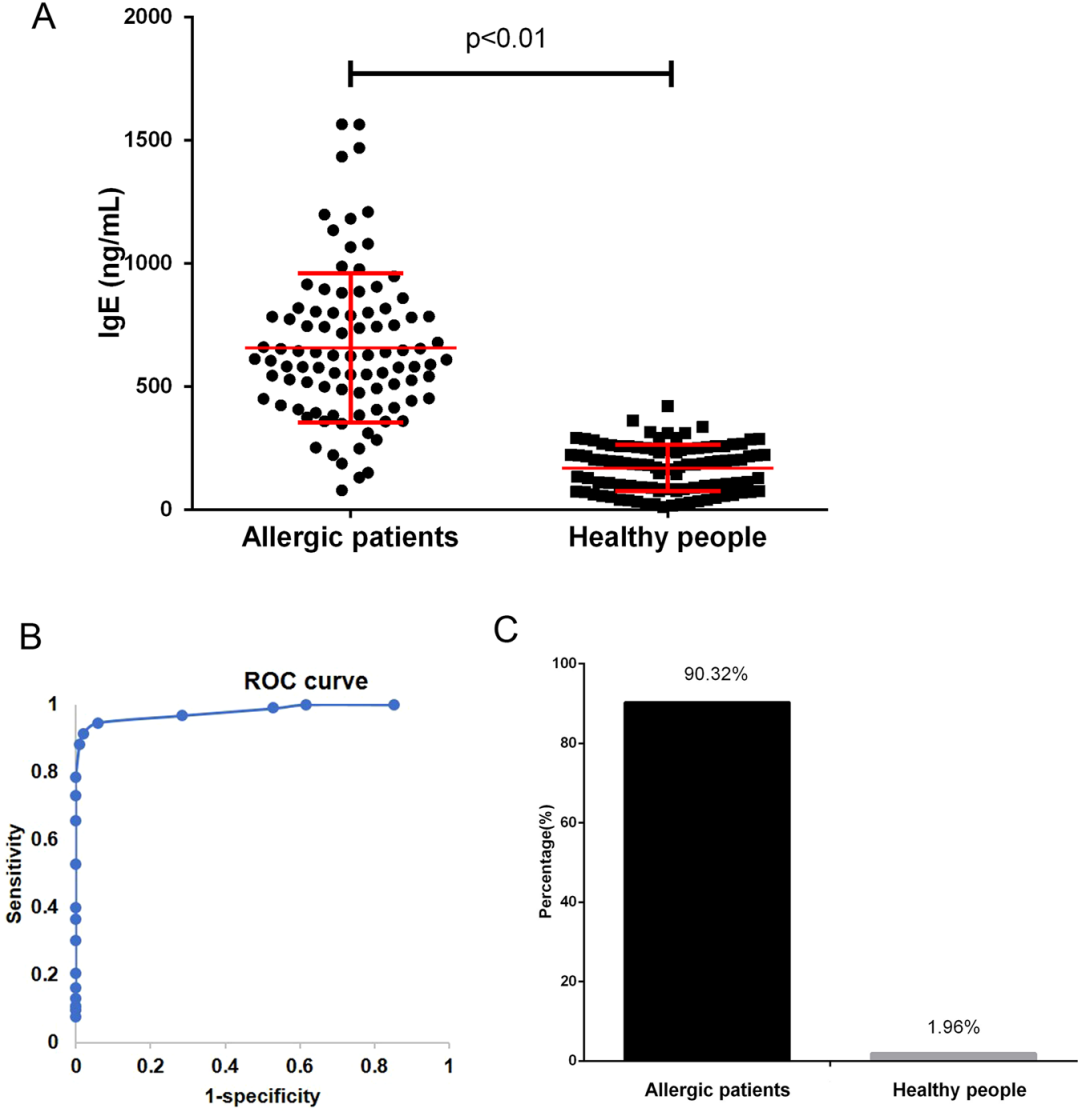
Peripheral blood total IgE is recommended for allergy diagnosis. (**A**) The peripheral blood total IgE (plasma IgE+ cell-bound IgE) of each sample in both allergic patients and healthy people; (**B**) a ROC curve was used to evaluate the diagnostic value of total IgE in allergic diseases; and (**C**) the percentages of allergic patients and healthy people diagnosed with allergy using 350 ng/mL peripheral blood total IgE as the cut-off value are shown.

At present, the cut-off value for plasma/serum IgE levels as a clinical diagnostic indicator of allergy is 240 ng/ml; however, as low as 33.57% of the allergic patients were diagnosed with allergy using this indicator. However, using 240 ng/ml cell-bound IgE as a cut-off value, 60.00% of the allergic patients were diagnosed with allergy (Fig. 3C). We analyzed the peripheral blood total IgE level of each sample (plasma IgE + cell-bound IgE), as shown in Fig. 4A, and the total IgE levels of the allergic patients were significantly higher than those of the healthy people (p < 0.01). A receiver operator characteristic curve (ROC curve) was used to evaluate the diagnostic value of total IgE in allergic diseases (Fig. 4B). The area under the curve (AUC) of total IgE for allergic diseases was 0.982, the standard error was 0.01, and the maximum value of sensitivity + specificity was 1.89. Therefore, we recommended 350 ng/mL peripheral blood total IgE as the cut-off value. Using this indicator, 90.32% of the allergic patients were diagnosed with allergy, while only 1.96% of healthy people were diagnosed with allergy (Fig. 4C). To test the accuracy of the cut-off value (350 ng/mL peripheral blood total IgE) for allergy diagnosis, we collected samples from another 50 allergic patients and 50 healthy people; 92.0% of the allergic patients had a peripheral blood total IgE level >350 ng/mL, while only 2.0% of the healthy people had a peripheral blood total IgE level >350 ng/mL (data not shown).

### Values of IgE receptors and IgE in both soluble and membrane-bound forms

We measured the concentrations of the soluble forms of the two IgE receptors in the samples by ELISA and found that the soluble FcɛRI (sFcɛRI) values in the allergic patients were significantly higher than those in the healthy people (Table 2). The sCD23 values in the allergic patients were also significantly higher than those in the healthy people. These soluble IgE receptor levels correlated with disease severity. We also measured FcεRI and CD23 expression on the surface of peripheral blood mononuclear cells (PBMCs) by flow cytometry. As shown in Table 2, the mean relative fluorescence intensity (RFI) of membrane-bound FcεRI (mFcεRI) in the allergic patients was significantly higher than that in the healthy people, and the membrane-bound CD23 (mCD23) values in the allergic patients were also significantly higher than those in the healthy people. The expression of these membrane-bound IgE receptors correlated with serum IgE values. IgE receptors could potentially be used as markers for the diagnosis of allergy. IgE receptors are potential *in vivo* modulators of IgE-mediated immune responses and are thus important for our basic understanding of allergic responses.

**Table 2:** Values of IgE receptors and IgE in both soluble and membrane-bound forms.

	sFcεRI (ng/mL)	mFcεRI (RFI)	sCD23 (ng/mL)	mCD23 (RFI)	Cell-bound IgE (ng/mL)	Plasma IgE (ng/mL)	Total IgE (ng/mL)
Allergic patients (mean ± SD)	2.41 ± 0.98	31,487 ± 14,271	2.20 ± 0.46	25,656 ± 8,601	409.6 ± 15.36	210.4 ± 16.48	658.2 ± 31.36
Healthy people (mean ± SD)	1.19 ± 0.85	15,567 ± 6,687	1.77 ± 0.21	11,490 ± 3,217	110.8 ± 6.152	46.68 ± 3.834	169.8 ± 9.313

sFcεRI, soluble FcεRI; mFcεRI, membrane-bound FcεRI; sCD23, soluble CD23; mCD23, membrane-bound CD23; RFI, relative fluorescence intensity.

## Discussion

IgE plays a critical role in immediate-type allergic reactions. Most somatic IgE is bound by its receptors^17^, and the serum IgE concentration does not accurately reflect the levels of systemic IgE^18,19^. At present, the current diagnostic method detects both allergen-specific IgE and total IgE levels, which is poorly correlated with allergy determination^20^. A new method is needed to investigate the IgE pool in the peripheral blood. There have been some reports on measuring human cell-bound IgE levels using flow cytometry^12^. Cell-bound IgE levels are calculated using the percentages of FcεRI^+^IgE^+^ and FcεRII^+^IgE^+^ cells among all whole-blood leukocytes, with the percentage of IgE^+^ cells in the receptor-bearing cell population multiplied by the mean fluorescence intensity (MFI) of IgE to generate a unit of IgE/cell. This reported method is simple and convenient, and the percentage of IgE^+^ cells reflects the cell-bound IgE level. However, this method does not quantitatively analyze the distribution of the whole IgE pool in the blood. In this method, serum IgE is detected by ELISA, while cell-bound IgE is detected by flow cytometry. Other defects in this method are that the MFI of IgE cannot be converted into the actual content unit (ng/mL) and that quantitative standards are lacking.

sFcɛRI has been reported to be a biomarker for IgE-mediated diseases^13^. We measured the concentrations of the soluble forms of the two IgE receptors in samples by ELISA and found that the sFcɛRI values in allergic patients were significantly higher than those in healthy people (Table 2). The sCD23 values in allergic patients were also significantly higher than those in healthy people. These soluble IgE receptors correlated with disease severity. sFcɛRI was reported to be a biomarker for IgE-mediated diseases, the titers of sFcɛRI in atopic individuals were significantly higher than those in controls, and the sFcɛRI levels and IgE levels correlated positively in all patients. sFcɛRI is mostly detected as a complex with IgE in the circulation, and more study is needed to assess the clinical implications of sFcɛRI^21^. sCD23 and galectin-3 have also been reported to be biomarkers that can be used to assess the severity of allergies, but the correlative data remain controversial^22^.

Regina Selb *et al*. investigated mCD23 and found that the mCD23 density on B cells of allergic patients was correlated with allergen-specific IgE levels and that mCD23 determined allergen uptake and subsequent T cell activation^8^. We measured FcεRI and CD23 expression on the surface of PBMCs by flow cytometry. As shown in Table 2, the mean RFI of mFcεRI in allergic patients was significantly higher than that in healthy people, and the mCD23 values in allergic patients were also significantly higher than those in healthy people. The expression of these membrane-bound IgE receptors correlated with IgE values in the serum. IgE receptors could potentially be used as markers for the diagnosis of allergy. IgE receptors are potential *in vivo* modulators of IgE-mediated immune responses and are thus important in our basic understanding of allergic responses.

The skin prick test is the gold standard for the diagnosis of allergy and is used to confirm allergic sensitization to suspected allergens and provide guidance for the treatment of patients. However, this test can be uncomfortable for patients and has an occasional risk of infection, though it is relatively safe. The measurement of allergen-specific IgE concentrations is very important, and the total number of available allergenic molecules has reached a diagnostically useful level; however, more molecules are needed to cover all clinically important allergen specificities^11^. Not all allergens that are in extracts have been defined at the molecular level yet. Other allergens have been well characterized but have not been produced at the quality level required for component-resolved diagnostic tests.

The advantages of evaluating at total IgE levels are that IgE antibodies indisputably play a key role in determining the allergen specificity of allergic disease and IgE responses in allergic individuals induced by allergen exposure have been shown to be rapid in previous studies. Allergen-specific IgE is the causative agent of allergic disease, and serum IgE levels generally correlate with the severity of allergic diseases. IgE level detection is also important for evaluating the therapeutic effect of monoclonal anti-IgE antibodies, such as omalizumab. Our new recommended method is not only simple but also convenient to carry out because it uses a kit already used in the clinic. This method is simple to perform and can be used in the analysis of large numbers of clinical samples.

The Elecsys II immunoassay is an aid in the diagnosis of allergy. As IgE is important in allergies, elevated IgE concentrations can be found in patients with allergic diseases such as hay fever, atopic bronchitis and dermatitis. Normal IgE values do not, however, mean that an allergic disease can be ruled out. For this reason, the quantitative determination of serum IgE concentrations for clinical differentiation between atopic and nonatopic diseases is useful only in combination with other clinical findings. The Elecsys IgE II assay uses monoclonal antibodies specifically directed against human plasma IgE and is used in hospitals. In our study, cell-bound IgE was dissociated from blood cells with lactic acid, and the released IgE could be detected by using the Elecsys IgE II assay. Dissociated cell-bound IgE and plasma IgE levels were detected using the same kit at the same time. Elevated total IgE levels aren’t exclusively found in allergic individuals, for example, indivuals bearing a parasitic infestation (helminths) also show elevated total IgE values. These patients would be diagnosed as allergic by using our method. In this particular case, only the detection of helminth- specific or allergen-specific IgE together with an analysis of patient history would be useful to give an adequate diagnosis.

In our study, all the cell-bound IgE was dissociated from blood cells, and the dissociated cell-bound IgE and plasma IgE levels were detected using the same ELISA kit at the same time; therefore, the data are consistent, and the units for both measurements are ng/mL. Our new recommended method is not only simple but also convenient to carry out because it uses a kit already used in the clinic. Any IgE detection kits used in hospitals can be applied to detect cell-bound IgE levels with the addition of several pretreatment steps. This method is simple to perform and can be used in the analysis of large numbers of clinical samples.

## Supplementary information

Supplementary Information.
