# Supplementary material for: Cell-bound IgE and plasma IgE as a combined clinical diagnostic indicator for allergic patients
Source: Sci Rep. 2020 Mar 13;10:4700. doi: 10.1038/s41598-020-61455-8 (PMC7069990; doi:10.1038/s41598-020-61455-8)
Supplement: Supplementary file 1 — Supplementary Information. [file 41598_2020_61455_MOESM1_ESM.pdf]

Cell-bound IgE and plasma IgE as a combined clinical diagnostic indicator for allergic patients

Chuanghua Qiu[1, #], Lihong Zhong[1, #], Chunxiu Huang[1], Jia Long[1,] Xuejun Ye[1,2], Jingbo Wu[1], Wenjie Dai[3], Wei Lv[4], Chongwei Xie[3, *], Junfang Zhang[1, *]

Supplementary table S1 The readout for the EUROLINE kit

Class	Concentration [kU/l]	Result
0	< 0.35 kU/l	No specific antibodies detected.
1	0.35–0.7 kU/l	Very low antibody titer, frequently no clinical symptoms when sensitization is present.
2	0.7–3.5 kU/l	Low antibody titer, existing sensitization that frequently occurs with clinical symptoms in the upper range of the class.
3	3.5–17.5 kU/l	Significant antibody titer, clinical symptoms usually present.
4	17.5–50 kU/l	High antibody titer, almost always with clinical symptoms.
5	50–100 kU/l	Very high antibody titer.
6	> 100 kU/l	Very high antibody titer.

Supplementary table S2 Symptoms of allergic patients

Number	Sex	Age	Main symptoms	Other symptoms
1	Female	35	Urticaria	Allergic Eczema
2	Male	23	Food Allergy	
3	Female	30	Allergic Rhinitis	Allergic Dermatitis
4	Male	22	Allergic Eczema	
5	Female	23	Food Allergy	
6	Male	27	Allergic Rhinitis	Urticaria
7	Female	20	Allergic Eczema	
8	Male	24	Urticaria	
9	Female	26	Allergic Rhinitis	
10	Male	22	Allergic Eczema	
11	Female	25	Asthma	Food Allergy
12	Male	20	Allergic Rhinitis	
13	Female	23	Allergic Dermatitis	
14	Male	32	Allergic Eczema	Allergic Rhinitis
15	Female	18	Allergic Rhinitis	
16	Male	22	Urticaria	

17	Female	18	Allergic Rhinitis	Urticaria
18	Male	23	Allergic Rhinitis	
19	Female	15	Allergic Dermatitis	Allergic Eczema
20	Male	35	Urticaria	
21	Female	12	Allergic Eczema	
22	Male	26	Urticaria	Food Allergy, Allergic Rhinitis
23	Female	23	Allergic Rhinitis	
24	Female	23	California Disease	
25	Male	25	Allergic Dermatitis	
26	Male	43	Urticaria	
27	Female	36	Food Allergy	Urticaria
28	Female	23	Allergic Eczema	
29	Male	34	Allergic Dermatitis	Allergic Rhinitis
30	Female	23	Allergic Rhinitis	
31	Male	52	Food Allergy	Food Allergy
32	Male	13	Urticaria	
33	Female	29	Asthma	Urticaria
34	Male	31	Food Allergy	
35	Male	43	Allergic Rhinitis	
36	Female	19	Allergic Dermatitis	
37	Male	20	Urticaria	
38	Female	37	Allergic Eczema	Food Allergy
39	Female	56	Food Allergy	Allergic Rhinitis
40	Male	23	Allergic Rhinitis	
41	Male	27	Allergic Dermatitis	
42	Female	35	Urticaria	
43	Male	28	Allergic Rhinitis	
44	Male	18	Allergic Dermatitis	
45	Male	22	Food Allergy	
46	Female	28	Allergic Rhinitis	
47	Male	27	Allergic Eczema	Urticaria
48	Female	25	Urticaria	
49	Female	27	Allergic Rhinitis	
50	Male	22	Urticaria	
51	Male	21	Allergic Rhinitis	
52	Female	27	Allergic Dermatitis	Allergic Rhinitis
53	Male	28	Food Allergy	Urticaria
54	Female	37	Allergic Dermatitis	
55	Male	12	Allergic Rhinitis	
56	Female	28	Urticaria	
57	Female	20	Allergic Dermatitis	
58	Male	32	Food Allergy	Food Allergy
59	Female	5	Allergic Eczema	
60	Male	19	Allergic Rhinitis	

61	Female	36	Allergic Eczema	
62	Female	39	Food Allergy	
63	Male	32	Allergic Rhinitis	
64	Male	43	Urticaria	
65	Female	38	Asthma	Allergic Rhinitis
66	Female	48	Urticaria	
67	Female	29	Allergic Rhinitis	
68	Male	6	Allergic Dermatitis	
69	Male	42	Food Allergy	
70	Female	27	Urticaria	Allergic Eczema
71	Female	32	Food Allergy	
72	Male	28	Urticaria	
73	Female	26	Allergic Dermatitis	
74	Male	52	Food Allergy	Food Allergy
75	Female	53	Food Allergy	
76	Female	26	Urticaria	
77	Male	42	Allergic Rhinitis	
78	Female	42	Food Allergy	Allergic Rhinitis
79	Female	52	Food Allergy	
80	Male	27	Allergic Rhinitis	
81	Female	40	Allergic Rhinitis	
82	Male	32	Food Allergy	
83	Female	45	Allergic Eczema	Urticaria
84	Female	46	Food Allergy	
85	Male	39	Food Allergy	
86	Female	21	Insect Venom Allergy	
87	Male	24	Urticaria	
88	Female	30	Food Allergy	Allergic Eczema
89	Female	40	Allergic Rhinitis	
90	Male	33	Food Allergy	
91	Male	26	Insect Venom Allergy	
92	Male	46	Allergic Eczema	
93	Female	13	Allergic Rhinitis	Food Allergy

Figure S1

The information on allergen-specific IgE sensitivities of the patients was analyzed, and the percentages of patients with different numbers of sensitivities indicated by allergen-specific IgEs were shown (A). The correlation between plasma IgE levels and cell-bound IgE levels is depicted as two overlapping Gaussian curves (B and C).

Figure S1

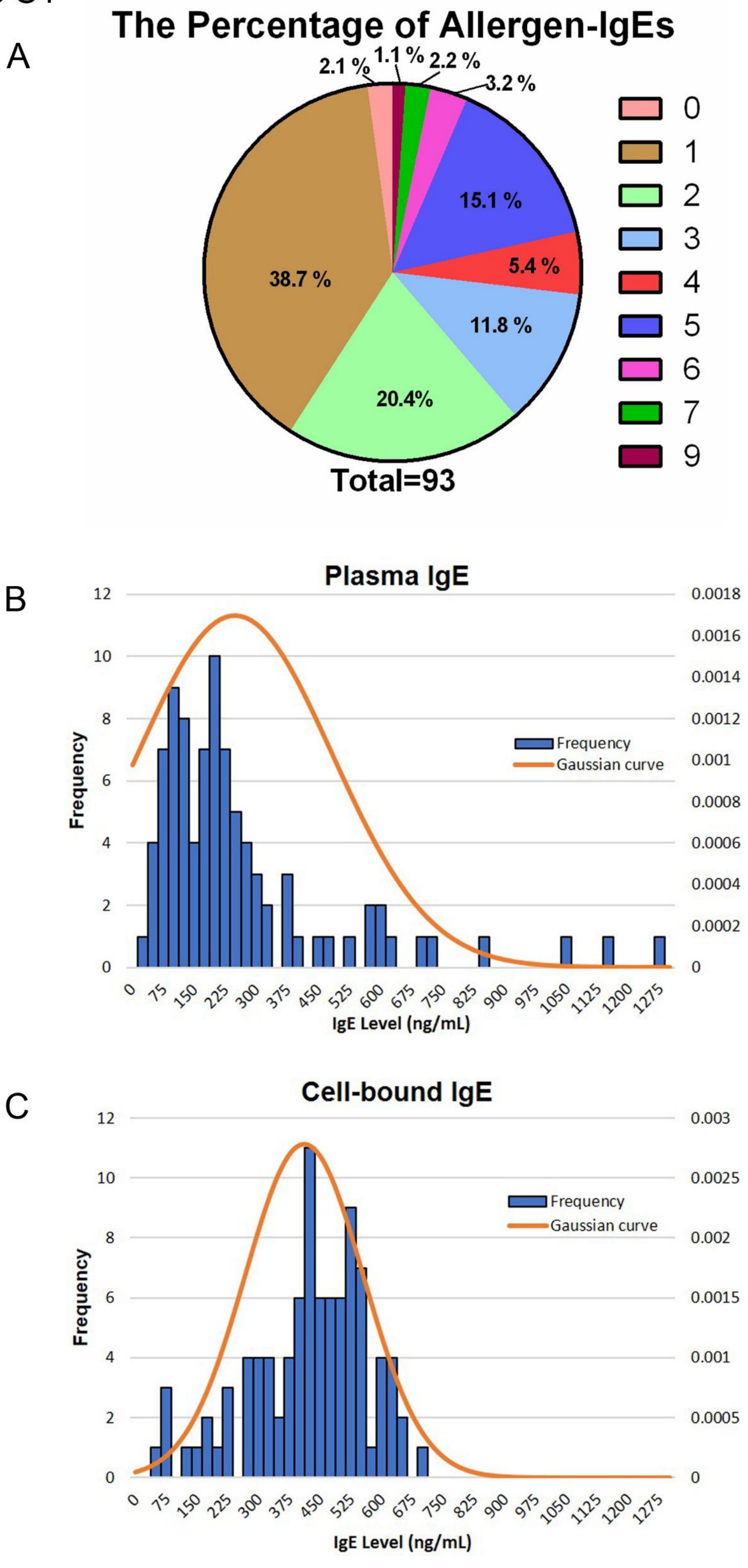
A
The Percentage of Allergen-IgEs
2.1 %
1.1 %
2.2 %
3.2 %
15.1 %
5.4 %
38.7 %
11.8 %
20.4%
Total=93
0
1
2
3
4
5
6
7
9
B
Plasma IgE
Frequency
Gaussian curve
IgE Level (ng/mL)
C
Cell-bound IgE
Frequency
Gaussian curve
IgE Level (ng/mL)
